# The impact of COVID-19 on clinical care, self-management, and mental health of patients with inflammatory arthritis

**DOI:** 10.1093/rap/rkab095

**Published:** 2021-12-04

**Authors:** Melissa Sweeney, Lewis Carpenter, Savia de Souza, Hema Chaplin, Hsiu Tung, Emma Caton, James Galloway, Andrew Cope, Mark Yates, Elena Nikiphorou, Sam Norton

**Affiliations:** 1 Health Psychology Section, Institute of Psychiatry, Psychology and Neuroscience, King’s College London, London, UK; 2 Centre for Rheumatic Diseases, King’s College London, London, UK

**Keywords:** Inflammatory arthritis, lockdown, COVID-19, clinical care, management, mental health, depression

## Abstract

**Objectives:**

The COVID-19 lockdown and ongoing restrictions in the UK affected access to clinical care, self-management, and mental health for many patients with Inflammatory Arthritis (IA). This study aimed to determine the impact of lockdown on IA clinical care, self-management, disease outcomes, and mental health.

**Methods:**

In total, 338 people with IA participated in a prospective study completing a series of online questionnaires. The questionnaires assessed demographics, IA condition and management, clinical care, quality of life, and mental health. Visual analogue scales (VAS) were completed at each assessment. Linear regression, controlling for confounders, was conducted to determine factors associated with physical and mental health outcomes.

**Results:**

Over half of participants reported worsening VAS by more than 10 points for Patient Global Assessment (PGA), pain, fatigue, and emotional distress during the initial lockdown. Changes in clinical care were associated with worse PGA (b = 8.95, *p* = 0.01), pain (b = 7.13, *p* = 0.05), fatigue (b = 17.01, *p* < 0.01) and emotional distress (b = 12.78, *p* < 0.01). Emotional distress and depression were also associated with worse outcomes in PGA, pain, and fatigue, while loneliness was not. In contrast, physical activity seemed to mitigate these effects. Loneliness did not show any associations with outcomes. Over time, these effects decreased or disappeared.

**Conclusions:**

Changes to clinical care due to lockdown were associated with worse disease outcomes in patients with IA. There has been a clear impact on mental health as well, with possibly complex relationships between mental health and psychosocial factors. Physical activity emerged as a key influence on disease outcomes and mental health.

**Key Messages:**

- The majority of patients reported worsened physical and mental health during lockdown.
- Changes in care and management were associated with worsening physical and mental health.
- The impact of lockdown changes on physical and mental health lessened over time.

## Introduction

Inflammatory arthritis (IA) is a collection of chronic autoimmune diseases requiring ongoing pharmacological treatment and careful adherence to self-management behaviours (1,2). The COVID-19 lockdown in the UK from March to July 2020 disrupted clinical care and required a period of self-isolation for many patients (3). Research into the impacts of changes to clinical care due to lockdown on IA patients' disease outcomes were needed in the UK as disruptions to daily routines caused by lockdown and ongoing restrictions could potentially alter self-management behaviours and disease outcomes.

This could also likely impact mental health, given that worse disease activity has been shown to be associated with worse mental health in IA (9,10). There is already some evidence that individuals with pre-existing physical or psychiatric comorbidities appear to be at higher risk of mental health consequences from the pandemic (15). As IA patients already have higher rates of comorbid mental health disorders compared to the general population (10,16), they could be particularly vulnerable.

Finally, since vulnerable IA patients were advised to self-isolate for 12 weeks to reduce their risk of contracting COVID-19 (known as 'shielding'), they could be at higher risk of mental health consequences from social isolation (3). In the general population, quarantining was shown to be a risk factor for both short- and long-term negative psychological effects such as increased rates of depression, insomnia, Post-traumatic Stress Disorder (PTSD), and substance abuse (17). Thus, research into the effects of social isolation on IA patients' physical and mental health was needed.

The objectives of this study were threefold. Firstly, to evaluate how the COVID-19 lockdown from March-July 2020 impacted patients' IA symptoms, self-management, and mental health in the short-term during a period of easing of initial lockdown restrictions in June/July 2020. Secondly, to evaluate the medium-term impacts on physical and mental health symptoms through November 2020. Thirdly, to determine the degree to which impacts on treatment and self-management were associated with worse physical and mental health symptoms in the short- and medium-term.

## Methods

Design & Recruitment:

The IA-COVID study is a longitudinal mixed-methods study examining the impact of COVID-19 on the quality of life of people with IA. Participants were recruited via social media and relevant charities. All participants provided written informed consent. Eligibility criteria were: aged 18-years or older, living in the United Kingdom and with an IA condition. Although the eligibility criteria specified respondents must be resident in the UK, three respondents from crown dependencies that form part of the British Isles but are not in the UK were included in the analyses. Ethical approval was obtained from King's College London Research Ethics Committee (LRS-19/20-18186). Written informed consent was obtained from all participants. The study complies with the Declaration of Helsinki.

The data included in the present analysis consisted of the baseline data collected between 1st June and 3rd July 2020, as lockdown restrictions in the UK were eased but shielding was ongoing. At the time of the baseline collection, shops re-opened, socializing with up to 6 people was allowed, and national travel resumed. Data from two additional follow-ups approximately 3-months apart were also collected. The first follow-up collected data from early September 2020, during another period of looser restrictions that included working from home, a curfew, and six-person limit on social gatherings. The second follow-up occurred in late November 2020, during a renewed period of strict restrictions, in which people were instructed to stay home except for essential trips. Two more follow-ups are planned for February 2021 and June 2021, however that data was not collected in time for the present study, but will be included in future analyses. Subsamples of participants also included an ecological momentary assessment study and a qualitative study (19).

Measures

The questionnaires assessed various aspects of the impact of the COVID-19 pandemic and lockdown measures between 23rd March and November 2020. Changes in these factors from before the lockdown were also evaluated.

The questionnaires were composed of the following full or shortened questionnaires: Demographics, IA condition, VAS disease activity scale, VAS pain scale, VAS emotional distress scale, Musculoskeletal Health Questionnaire (MSKHQ), PHQ-8, Generalised Anxiety Disorder Assessment (GAD-7), UCLA Loneliness scale, Lubben Social Networks Scale, Healthy Eating Assessment, Sleep Questionnaire, Global Physical Activity Questionnaire (GPAQ), COM-B. Some questions were modified to clarify them in the context of COVID-19. Additional researcher-designed questions were included regarding: IA management, changes to medication (beyond those recommended by clinical care team), changes (yes/no) to various areas of clinical care, changes in self-management, comorbidities, food shortages, social contact satisfaction, COVID-19 experience and symptoms, COVID-19 attitudes, fear of COVID-19. and the impact of COVID-19 on employment, finances, and general wellbeing. Not all of these measures were used in the present analyses, but may be used in sub-studies or futures analyses.

**Disease outcome measures:**

Visual Analog Scales (VAS) were completed for the previous week and all ranged from 0-100. The baseline study also retrospectively assessed pre-lockdown and early lockdown for the Patient Global Assessment (PGA), pain, and fatigue. VAS are considered appropriate to measure the intensity of an experience, such as distress or pain (20), and have been shown to have good validity and reliability (21,22).

**Lifestyle measures:**

Diet was evaluated by a shortened Healthy Eating Assessment measuring inflammatory diet patterns (23), which has good validity and sensitivity (24). Higher scores indicated a more inflammatory diet. The baseline questions asked about frequency of inflammatory and other foods eaten (fried/fast foods, sweets, sweetened beverages, fruit, vegetables, dairy, and red or processed meats) and also asked if they were eating less, the same, or more of each item compared to before the COVID-19 measures.

Physical activity was measured at baseline with one question modified from the MSKHQ asking "On how many days did you do a total of 30 minutes or more of physical activity, which was enough to raise your heart rate?". Additionally, the baseline questionnaire asked if they engaged in less, the same, or more physical activity compared with before the lockdown. The MSKHQ also shows good validity and reliability (25). Finally, one researcher-designed question regarding changes to medication (yes/no to changes in dosage and/or frequency) was included.

**Quality of Life and Mental Health measures:**

Emotional distress was measured with a VAS for the previous week in the baseline ("post-lockdown") in June/July 2020 and both follow-up questionnaires. The baseline questionnaire also asked about emotional distress retrospectively for pre-lockdown (early March 2020) and peri-lockdown (April 2020). The Personal Health Questionnaire Depression Scale (PHQ-8) was used to measure depressive symptoms and has been validated in many contexts (26). Two questions from the Generalized Anxiety Disorder Assessment (GAD-7) were used to assess anxiety: "Feeling nervous, anxious, or on edge" and "Not being able to stop or control worrying". The GAD-7 has shown good reliability and validity (27). Several researcher-designed questions about psychosocial concerns were included. A shortened version of the UCLA Loneliness scale using 4 questions relevant to lockdown context was used to assess loneliness in the baseline questionnaire. This scale has been established as a reliable and valid measure of loneliness (28). One researcher-designed question assessed the level of fear or concern participants felt about COVID-19 ("How concerned do you feel about COVID-19?").

<u>Statistical analysis:</u>

Changes in mean VAS and standard deviations were calculated for PGA, pain, fatigue, and emotional distress. Clinically meaningful improvement or worsening in each VAS score was considered as a change of 10-points or more from pre-lockdown to post-lockdown in June/July

2020 (29). Repeated measures ANOVA were run for to determine if there was any difference over time for PGA, pain, fatigue, emotional distress, diet, physical activity, depression, loneliness, or fear of COVID-19. Additionally, T-tests were conducted to assess the difference between scores at the different timepoints. The percent of the sample reporting better, same, or worse outcomes compared to before lockdown on the VAS and the 95% confidence intervals were calculated for PGA, pain, fatigue, and emotional distress for pre- to post-lockdown scores, and for changes in clinical care and self-management behaviours. Violin plots of these changes in VAS scores were also produced. Demographics and key clinical characteristics were compared for participants who completed all surveys with those who dropped out.

Finally, linear regressions controlling for potential confounders of age, gender, condition, disease duration, and pre-lockdown disease activity or emotional distress, were conducted to determine the factors associated with worse outcomes: (1) on physical health measures and (2) in mental health. First, baseline (June/July 2020) changes in clinical care, changes in medication, inflammatory diet, and physical activity were used as predictors of PGA, pain, fatigue, and emotional distress at baseline, September, and November follow ups. Changes in clinical care and medication were used as categorical predictors, where clinical care was coded as yes/no for each area of care which may have been affected while medication changes were coded as yes/no, but could include changes to either dosage or frequency. The remaining factors were used as continuous variables. Next, the baseline (June/July 2020) mental health factors of emotional distress, depression, and loneliness were used as continuous predictors of PGA, pain, and fatigue at baseline, September, and November follow ups. Models were completed separately for baseline, September, and November outcomes. Effect sizes at all time points were calculated using omega-squared. All analyses were carried out using Stata (ver 16.0).

## **Results**

Table 1 summarizes the baseline characteristics of the sample by IA condition. A total of 338 participants completed the baseline assessment in June. Data were available for 203 (60.0%) and 173 (51.2%) participants at the September and November follow-ups, respectively. The sample was largely female (90.2%) and white (97.5%), with an average age of 47.9 years old (range 19-77 years). Figure 1 shows a flowchart of the recruitment process. Those who completed all questionnaires were significantly older ($p<0.01$) and had significantly higher scores on baseline pain ($p<0.01$) compared with those who dropped out.

Repeated measures ANOVA found differences over time for PGA ($F(4, 1036) = 34.58$ $p<0.01$), pain ($F(4, 1036)= 40.54$, $p<0.01$), fatigue ($F(4, 1035)= 43.11$, $p<0.01$), emotional distress ($F(4, 1012) = 55.67$, $p<0.01$), diet ($F(2, 273)= 10.88$, $p<0.01$), depression ($F(2,286) =8.43$, $p<0.01$)), loneliness ($F(2, 281) = 5.97$, $p<0.01$), and fear of COVID-19 ($F(2,270) = 8.26$, $p<0.05$). Physical activity was not significantly different across timepoints ($F(2,262) = 0.15$, $p=0.86$). T-tests are shown in Table 1 identifying the timepoints with significant differences.

Physical Health:

The mean VAS scores and standard deviations during pre-, peri-, and post-lockdown from baseline and the September and November follow-ups are displayed in Table 1. On average, all measures of disease activity (PGA, fatigue, and pain) showed worsening from pre-lockdown (February 2020) to post-lockdown (June 2020) (Figure 2 & Supplementary Figure S1, available at *Rheumatology Advances in Practice* online.). In contrast, emotional distress was highest in peri-lockdown. The majority of the overall sample reported worsening outcomes during the lockdown for all disease measures; however, the results were mixed and many participants also reported that their disease activity stayed the same while a minority reported improvements.

At the follow-ups, the VAS scores for PGA, pain, fatigue, and emotional distress had improved relative to the end of the lockdown in June, but on remained higher than pre-lockdown levels. Pain and fatigue VAS scores showed consistent trends downward over time after the lockdown while PGA and emotional distress showed a slight increase again in November.

Demographic and earlier clinical measurements were examined for associations with later physical outcomes. None of the demographic characteristics were predictive of physical health outcomes at baseline or the follow ups except for duration of the IA condition, which was significantly associated with PGA in September (b=0.0, p<0.01). The pre-lockdown and post-lockdown measurements of PGA, pain, and fatigue were significantly associated with all of their respective measurements at baseline and/or follow-ups, with the exception of PGA pre-lockdown, which was not significantly associated with PGA in November.

Clinical Care:

Overall, 87.45% of participants experienced change to their clinical care (as indicated in Figure 3) with the greatest impact on clinical appointments (76.8%), GP appointments (59.1%), and blood tests (53.6%). Detailed breakdown of the percent [95% CI] of participants with any changes in each of the clinical care areas during the lockdown is provided in Supplementary Table S1, available at *Rheumatology Advances in Practice* online.

Linear regression analyses (Table 2) demonstrate that those reporting changes to clinical care at baseline had significantly worse PGA (b=8.95, p=0.01), pain (b=7.13, p=0.05), fatigue (b=17.01, p<0.01) and emotional distress (b=12.78, p<0.01) at baseline, even when controlling for pre-lockdown levels of the outcome. Results remained significant when controlling for fear of COVID-19 and COVID-19 infection status. The effect size of changes to clinical care were small for PGA ($w^2$= 0.02) pain ($w^2$ = 0.01), and emotional distress ($w^2$=0.03), whereas it was medium for fatigue ($w^2$=0.07).

At the follow-ups, the impact of clinical care changes only remained significant for fatigue in September (b= 10.76, p=0.04), but was no longer significant by November. The effect size for fatigue decreased over time with it having faded to a small effect size in September ($w^2$=0.02) compared to the medium effect size at baseline. None of the other outcomes of PGA, pain, and emotional distress remained significant over time at the follow ups.

Table 2 shows that, overall, the majority of participants (89.7%) reported not altering their medication during the lockdown period at baseline. Table 2 also shows the linear regressions for changes in medication, adjusted for pre-lockdown levels of outcomes. Medication non-adherence at baseline was also significantly associated with worse PGA (b=13.12, $p$<0.01), pain (b= 11.47, $p$<0.01), and fatigue (b=14.83, $p$<0.01), though not emotional distress (b=7.43, $p$<0.12) at baseline. Effect sizes for changes to medication were small for PGA ($w^2$=0.04), pain ($w^2$=0.03), and fatigue ($w^2$=0.04). None of these effects were still significant at the follow-ups in September or November.

When the analyses were repeated with only participants who completed all questionnaires, clinical care changes were no longer significant at baseline for emotional distress nor was it significant in September for pain or fatigue, but it was significant in November for pain (b=16.9, p=0.05). Changes in medication were no longer significant at baseline for fatigue.

<u>Lifestyle:</u>

Over half (64.3%) of participants reported making changes to their diet during the lockdown and 51.1% reduced their physical activity. Table 2 displays the regression coefficients for self-management behaviours as predictors of disease outcomes. Inflammatory diet was significantly associated with fatigue only (b=0.99, p=0.02), whereas physical activity was associated with PGA (b=-2.40, p<0.01), pain (b=-2.43, p<0.01), fatigue (b=-2.5, p<0.01), and emotional distress (b=-2.41, p<0.01) in June. Results remained significant when controlling for fear of COVID-19 and COVID-19 infection status. The effect sizes of physical activity were medium for PGA ($w^2$=0.07), pain ($w^2$=0.07), fatigue ($w^2$=0.06), and emotional distress ($w^2$=0.04).

At the follow ups, physical activity at baseline remained significantly associated with pain (b=-1.94, p= 0.01) and fatigue (b= -19.1, p=0.02) in September, while by November none of the effects remained. The effect sizes for physical activity also decreased over time for pain ($w^2$=0.04) and fatigue ($w^2$=0.03) by September compared to baseline. Inflammatory diet was no longer significant for fatigue at follow ups. However, while it was not significantly associated with PGA at baseline in June, was significant later in Nov it was significant later in November for PGA (b= 1.78, p<0.01, $w^2$=0.05), indicating a delayed effect which showed later in time. When the regressions were repeated with the sample including only those who completed all questionnaires, diet was significant at baseline for PGA (b=2.25, p=0.01) and pain (b=2.26, p=0.02). However, physical activity was no longer significant for pain at the September follow up.

Mental Health:

The majority (58.6%) of participants in the overall sample reported their emotional distress worsened during the lockdown, though changes were mixed (Supplementary Figure S1, available at *Rheumatology Advances in Practice* online.). This pattern was similar across conditions, with the exception of Juvenile Idiopathic Arthritis, but this group only had a sample size of 5.

Table 3 shows that emotional distress at the end of the lockdown was found to be significantly associated with PGA (b= 0.21, p<0.01), pain (b=0.24, p<0.01), and fatigue (b=0.36, p<0.01). Similarly, depression was also associated with all of the disease activity outcomes in June at the end of lockdown: PGA (b=0.95, p<0.01), pain (b=0.92, p<0.01), and fatigue (b=1.56, p<0.01). Loneliness was not associated with any of the disease activity outcomes. Results remained significant when controlling for fear of COVID-19 and COVID-19 infection status.

At the follow-ups, emotional distress remained significant for PGA (b=0.15, p=0.01), pain (b=0.14, p=0.01), and fatigue (b=0.14, p=0.02) in September. Depression also remained significant in September for PGA (0.65, p=0.04), pain (b=0.48, p=0.08), and fatigue (b=0.61, p=0.04), while in November it was significant only for PGA (b= 0.65, p =0.04) and fatigue (b=0.61, p=0.04).

When the regressions were repeated with the sample including only those who completed all questionnaires, emotional distress was no longer significant in the September follow up for PGA, pain, or fatigue and depression was no longer significant for PGA and fatigue. In November, depression was no longer significant for PGA or fatigue. None of the demographic characteristics were significantly associated with emotional distress at baseline or the follow ups. The pre-lockdown and post-lockdown measurements of emotional distress were significantly associated with the later measurements of emotional distress at baseline and follow-ups.

The effect sizes for emotional distress were large for PGA ($w^2$= 0.10), pain ($w^2$= 0.12), and fatigue ($w^2$= 0.23). For depression, the effect sizes were medium for PGA ($w^2$=0.09) and pain ($w^2$=0.07) and large for fatigue ($w^2$=0.17). The effect size for social contact on pain was small ($w^2$=0.02). The effect sizes for emotional distress were reduced at the follow-up in September: PGA ($w^2$=0.03), pain ($w^2$=0.03), and fatigue ($w^2$=0.02). For depression, the effect sizes were also reduced at follow up in both September: PGA ($w^2$=0.03), pain ($w^2$=0.05), and fatigue ($w^2$=0.04), and November: PGA ($w^2$=0.02) and fatigue ($w^2$=0.02).

## Discussion

Patients with IA experienced significant disruptions to their clinical care, lifestyle, and mental health during the COVID-19 lockdown and ongoing restrictions in 2020. These changes were associated with worse disease activity, indicating that clinicians should be aware of the adverse effects of changes to clinical care and consider ways to mitigate the negative effects.

Changes to lifestyle behaviours during the lockdown varied widely among patients. The mixed results for inflammatory diet over time could indicate differing short-term and long-term mechanisms, such as different inflammatory pathways or causes. Physical activity changes were also mixed, reflecting results in other studies (6,30,31). However, as physical activity had a larger impact on disease activity measures than changes in medication and clinical care in the long-term, its importance in IA self-management and future interventions is underscored. Physical activity may also offset some of the impacts of disruptions to clinical care so clinicians should continue to support patient education around it (33,34). The qualitative sub-study associated with this study lends further insight into explaining behavior changes (19).

It has already been established that, outside of lockdowns, emotional distress is intertwined with worse disease outcomes (35–37). Our results suggest that this is consistent under lockdown too (38). Other research has indicated mental health concerns have increased during the pandemic, suggesting it may be of increased importance during this time (39–41). This should prompt professionals to prioritize access to mental health resources to prevent emotional distress from affecting IA outcomes.

The null results for loneliness may be indicative of the overlap between different aspects of mental health and psychosocial factors. While this study did not find loneliness to be directly associated with physical health outcomes, other research has indicated that loneliness has worsened during lockdowns and has been associated with depression and suicidality (42,43). Additionally, the UCLA loneliness scale is a common measure, but has not been validated in the context of lockdowns so it should be interpreted with caution.

This study had the benefit of a large sample size, although it appears to have some bias in gender, age, and ethnicity. The study was also limited in that the pre-lockdown and peri-lockdown measures from baseline were retrospective self-report up to several months prior. The analyses included descriptive statistics of all of the retrospective measures, but the regressions were limited to more recent measures (last two weeks), which would be more reliable. Also, some of the questions were shortened from existing scales, modified to fit the context of COVID-19, or researcher-designed in the absence of pre-existing scales relevant to COVID-19. These questions would also be a limitation as they were not validated.

The current analyses suggest the impacts of lockdown show a general decrease over time. As there will be further follow-up questionnaires in February 2021 and June 2021, future analyses can potentially examine whether these decreases continue over longer periods of time.

Lastly, this study suggests that professionals should consider the adverse effects on patients of changes to care and lifestyle due to the COVID-19 lockdown and restrictions as these changes are associated with worsening of disease outcomes and mental health. Additionally, the decrease in the impacts over time indicate that more support during initial phases of lockdowns, followed by gradual easing, could be most appropriate. Guided by insights from this study, professionals have the potential to improve patient support in the future and prevent adverse impacts on patient outcomes.

## Acknowledgements

**Author contributions:** All of the authors were involved in drafting the article. SN, LC, MS, HC, SdS and LH were responsible for the study design. Acquisition of data, analysis, and interpretation was completed by MS and SN.

**Funding:** No specific funding was received from any bodies in the public, commercial or not-for-profit sectors to carry out the work described in this article.

**Disclosure statement:** The authors have declared no conflicts of interest.

**Data availability statement:** The data underlying this article will be shared on reasonable request to the corresponding author.

**Table 1. Sample characteristics**

| | Total Sample | P-value | Psoriatic Arthritis | Rheumatoid Arthritis | Ankylosing spondylitis | Connective Tissue Disease | Juvenile Idiopathic Arthritis |
|---|---|---|---|---|---|---|---|
| **N** | 338 | | 98 | 100 | 50 | 85 | 5 |
| **Age, mean (SD)** | 47.9 (13.6) | | 46.4 (12.0) | 53.1 (14.4) | 41.2 (12.1) | 48.7 (12.7) | 28.2 (11.5) |
| **Gender, %** | | | | | | | |
| Male | 9.2 | | 13.3 | 8 | 18 | 1.2 | 0 |
| Female | 90.2 | | 85.7 | 92 | 82 | 97.7 | 100 |
| Non-binary or other | 0.6 | | 1.0 | 0 | 0 | 1.2 | 0 |
| **Education, %** | | | | | | | |
| No formal qualifications | 3.6 | | 3.1 | 2 | 4 | 5.9 | 0 |
| O-level, GCSE or equivalent | 21.3 | | 23.5 | 22 | 16 | 21.2 | 20 |
| A-level or equivalent | 21.0 | | 25.5 | 23 | 16 | 16.5 | 20 |
| Undergraduate degree or equivalent | 32.3 | | 30.6 | 27 | 45 | 31.8 | 40 |
| Postgraduate degree or equivalent | 21.9 | | 17.4 | 26 | 18 | 24.7 | 20 |
| **COVID Infection** | | | | | | | |
| Post-lockdown (June) | 16.5% | | 12.6% | 20.6% | 12.5% | 19.5% | 0% |
| Follow-Up 3 Months (September) | 12.43% | | 12.0% | 18.5% | 5.6% | 8.2% | 0% |
| Follow-Up 5 Months (November) | 13.29% | | 17.9% | 14.5% | 5.3% | 12.2% | 0% |
| **Mean MSKHQ scores (SD)** | 33.9 (12.0) | | 35.5 (1.1) | 31.3 (1.2) | 37.5 (1.7) | 32.7 (1.4) | 39 (4.6) |
| **Medication, %** | | | | | | | |
| Biologics | 44.7% | | 52.0% | 55.0% | 72.0% | 89.4% | 80.0% |
| Traditional conventional disease-modifying antirheumatic drugs | 76.9% | | 78.6% | 79.0% | 50.0% | 88.2% | 80.0% |
| NSAIDs | 66.3% | | 78.6% | 61.0% | 78.0% | 50.6% | 80.0% |
| Steroids | 50.0% | | 46.9% | 53.0% | 48.0% | 51.8% | 40.0% |
| **Shielding, %** | 54.1% | | 46.2% | 53.2% | 55.6% | 65.0% | 25.0% |

| | | | | | | | |
|---|---|---|---|---|---|---|---|
| **Mean PGA (SD)** | | | | | | | |
| Pre-lockdown | 44.5 (23.7) | | 47.8 (23.5) | 41.4 (21.9) | 53.0 (22.3) | 38.7 (24.8) | 56.4 (26.7) |
| Peri-lockdown | 53.2 (24.7) | **P<0.01** | 55.5 (22.9) | 50.6 (24.8) | 61.9 (23.8) | 48.5 (25.7) | 52.4 (27.6) |
| Post-lockdown (June) | 57.7 (25.3) | **P<0.01** | 60.2 (23.0) | 53.8 (25.5) | 67.9 (21.4) | 52.9 (27.8) | 66.2 (26.3) |
| Follow-Up 3 Months (September) | 47.8 (25.6) | P=0.07 | 52.3 (25.6) | 43.8 (24.7) | 58.0 (23.7) | 44.4 (26.0) | 44.3 (38.8) |
| Follow-Up 5 Months (November) | 48.5 (25.2) | P=0.04 | 53.5 (24.2) | 43.9 (25.8) | 51.7 (22.0) | 47.1 (27.4) | 52.5 (5.0) |
| **Mean Pain (SD)** | | | | | | | |
| Pre-lockdown | 42.6 (25.6) | | 46.0 (26.5) | 40.1 (23.7) | 49.5 (26.0) | 36.2 (24.7) | 66.0 (27.0) |
| Peri-lockdown | 51.1 (26.0) | **P<0.01** | 52.8 (25.0) | 49.7 (26.5) | 59.0 (25.0) | 45.5 (26.1) | 65.0 (27.0) |
| Post-lockdown (June) | 56.7 (26.4) | **P<0.01** | 58.3 (25.0) | 54.8 (27.0) | 65.0 (23.5) | 50.7 (27.9) | 79.2 (9.3) |
| Follow- Up 3 Months (September) | 46.8 (25.5) | P=0.03 | 53.1 (25.3) | 40.1 (24.4) | 54.5 (24.3) | 44.3 (25.3) | 73.7 (14.8) |
| Follow-Up 5 Months (November) | 45.4 (24.8) | P=0.12 | 49.8 (24.8) | 42.0 (26.2) | 48.2 (22.0) | 42.1 (24.4) | 65.0 (12.9) |
| **Mean Fatigue (SD)** | | | | | | | |
| Pre-lockdown | 46.9 (26.2) | | 59.5 (25.9) | 42.3 (28.1) | 49.4 (23.3) | 46.6 (25.5) | 45.6 (25.6) |
| Peri-lockdown | 57.1 (25.8) | **P<0.01** | 61.4 (23.3) | 51.2 (29.3) | 59.8 (23.5) | 58.1 (24.6) | 48.2 (27.5) |
| Post-lockdown (June) | 61.4 (26.5) | **P<0.01** | 65.1 (22.8) | 55.2 (31.0) | 65.4 (24.0) | 61.7 (25.2) | 63.8 (31.6) |
| Follow- Up 3 Months (September) | 59.0 (26.2) | **P<0.01** | 61.8 (24.4) | 53.1 (28.3) | 68.9 (24.6) | 59.2 (24.9) | 70.0 (26.5) |
| Follow-Up 5 Months (November) | 54.1 (25.6) | **P<0.01** | 57.4 (20.4) | 47.8 (29.2) | 58.5 (24.1) | 56.5 (25.4) | 51.0 (34.1) |
| **Mean Emotional Distress (SD)** | | | | | | | |
| Pre-lockdown | 31.0 (26.3) | | 32.2 (27.0) | 27.7 (25.8) | 35.1 (27.7) | 31.0 (25.7) | 29.2 (18.1) |
| Peri-lockdown | 49.1 (29.1) | **P<0.01** | 48.9 (28.3) | 45.7 (29.5) | 58.0 (28.2) | 47.5 (29.4) | 56.0 (31.6) |
| Post-lockdown (June) | 48.8 (29.2) | P=0.55 | 49.0 (28.7) | 45.2 (28.8) | 56.4 (27.8) | 47.3 (29.7) | 62.2 (42.0) |
| Follow- Up 3 Months (September) | 38.9 (28.9) | **P<0.01** | 41.8 (27.7) | 33.5 (28.9) | 53.9 (26.7) | 36.5 (29.2) | 53.3 (25.2) |
| Follow-Up 5 Months (November) | 39.8 (27.6) | **P<0.01** | 42.0 (26.6) | 38.9 (31.3) | 40.1 (24.1) | 40.1 (26.1) | 24.5 (17.9) |
| **Inflammatory Diet** | | | | | | | |
| Pre-lockdown | -- | -- | -- | -- | -- | -- | -- |
| Peri-lockdown | -- | -- | -- | -- | -- | -- | -- |
| Post-lockdown (June) | 8.8 (3.0) | | 9.3 (0.3) | 8.5 (0.3) | 8.9 (0.4) | 8.4 (0.3) | 8.5 (1.5) |
| Follow- Up 3 Months (September) | 7.8 (2.4) | **P<0.01** | 8.3 (0.3) | 7.2 (0.3) | 9.4 (0.6) | 7.7 (0.4) | 7.3 (2.4) |
| Follow-Up 5 Months (November) | 7.8 (2.5) | P=0.83 | 8.0 (0.4) | 7.4 (0.4) | 8.6 (0.5) | 7.5 (0.4) | 8.8 (1.4) |

| | | | | | | | |
|---|---|---|---|---|---|---|---|
| **Physical Activity**<br>Pre-lockdown<br>Peri-lockdown<br>Post-lockdown (June)<br>Follow- Up 3 Months (September)<br>Follow-Up 5 Months (November) | --<br>--<br>2.8 (2.1)<br>3.1 (2.1)<br>3.2 (2.2) | --<br>--<br><br>P=0.79<br>P=0.3 | --<br>--<br>2.5 (0.2)<br>2.3 (0.3)<br>2.5 (0.3) | --<br>--<br>3.6 (0.3)<br>3.7 (0.3)<br>3.9 (0.3) | --<br>--<br>2.8 (0.3)<br>3.4 (0.5)<br>3.2 (0.6) | --<br>--<br>2.5 (0.2)<br>3.2 (0.4)<br>3.0 (0.4) | --<br>--<br>2.0 (1.0)<br>2.0 (1.0)<br>1.5 (0.3) |
| **Depressive Symptoms**<br>Pre-lockdown<br>Peri-lockdown<br>Post-lockdown (June)<br>Follow- Up 3 Months (September)<br>Follow-Up 5 Months (November) | --<br>--<br>10.5 (6.1)<br>8.6 (5.9)<br>9.2 (5.9) | --<br>--<br><br>**P<0.01**<br>**P<0.01** | --<br>--<br>11.0 (0.6)<br>9.8 (0.9)<br>10.6 (0.9) | --<br>--<br>8.9 (0.7)<br>7.1 (0.8)<br>7.4 (0.8) | --<br>--<br>12.0 (0.9)<br>12.5 (1.4)<br>10.6 (1.3) | --<br>--<br>10.9 (0.6)<br>7.9 (0.9)<br>9.8 (0.9) | --<br>--<br>12 (4.1)<br>9.3 (4.4)<br>8 (2.9) |
| **Loneliness**<br>Pre-lockdown<br>Peri-lockdown<br>Post-lockdown (June)<br>Follow- Up 3 Months (September)<br>Follow-Up 5 Months (November) | --<br>--<br>10.3 (4.0)<br>9.9 (3.8)<br>9.3 (4.0) | --<br>--<br><br>**P<0.01**<br>**P=0.04** | --<br>--<br>10.2 (0.4)<br>10.0 (0.6)<br>9.1 (0.6) | --<br>--<br>10.4 (0.4)<br>9.7 (0.5)<br>9.3 (0.6) | --<br>--<br>10.8 (0.6)<br>12.1 (0.8)<br>9.9 (0.9) | --<br>--<br>10.1 (0.5)<br>8.9 (0.6)<br>9.3 (0.7) | --<br>--<br>11.0 (2.1)<br>13 (1.5)<br>8.8 (1.3) |
| **COVID fear**<br>Pre-lockdown<br>Peri-lockdown<br>Post-lockdown (June)<br>Follow- Up 3 Months (September)<br>Follow-Up 5 Months (November) | --<br>--<br>3.8 (1.0)<br>3.4 (1.0)<br>3.5 (1.0) | --<br>--<br><br>**P<0.01**<br>**P=0.01** | --<br>--<br>3.7 (0.1)<br>3.4 (0.2)<br>3.5 (0.1) | --<br>--<br>3.7 (0.1)<br>3.4 (0.2)<br>3.5 (0.1) | --<br>--<br>4.0 (0.1)<br>4.0 (0.2)<br>4 (0.2) | --<br>--<br>3.7 (0.1)<br>3.3 (0.2)<br>3.6 (0.2) | --<br>--<br>4.0 (1.0)<br>3.7 (0.3)<br>2.5 (0.3) |

[a]*SD:standard deviation. PGA: patient global assessment; MSKHQ: musculoskeletal health questionnaire. Bold text indicates significant results.*

**Table 2. Adjusted regression coefficients for clinical care and lifestyle.**

| | PGA | p | Pain | p | Fatigue | p | Emotional Distress | p |
|---|---|---|---|---|---|---|---|---|
| **Baseline in June** | | | | | | | | |
| Changes to clinical care | 8.95 | **0.01** | 7.13 | **0.047** | 17.01 | **<0.01** | 12.78 | **<0.01** |
| Changes to medication | 13.13 | **<0.01** | 11.47 | **<0.01** | 14.83 | **<0.01** | 7.43 | 0.12 |
| Inflammatory Diet | 0.61 | 0.10 | 0.72 | 0.19 | 0.99 | **0.02** | 0.88 | 0.08 |
| Physical activity | -2.40 | **<0.01** | -2.43 | **<0.01** | -2.50 | **<0.01** | -2.41 | **<0.01** |
| **Follow-Up September** | | | | | | | | |
| Changes to clinical care | 5.34 | 0.29 | 7.96 | 0.09 | 10.76 | **0.04** | 5.88 | 0.30 |
| Changes to medication | 2.45 | 0.65 | 1.97 | 0.70 | 3.09 | 0.57 | 0.24 | 0.97 |
| Inflammatory Diet | -0.33 | 0.53 | -0.27 | 0.58 | 0.18 | 0.73 | -0.36 | 0.55 |
| Physical activity | -1.25 | 0.11 | -1.94 | **0.01** | -19.10 | **0.02** | -0.61 | 0.48 |
| **Follow-Up November** | | | | | | | | |
| Changes to clinical care | 1.02 | 0.84 | 5.70 | 0.19 | -2.02 | 0.66 | -2.66 | 0.67 |
| Changes to medication | 7.35 | 0.20 | -1.50 | 0.76 | 6.39 | 0.21 | 7.64 | 0.29 |
| Inflammatory Diet | 1.78 | **<0.01** | 0.19 | 0.73 | 0.34 | 0.54 | 0.90 | 0.24 |
| Physical activity | -1.59 | 0.05 | -0.53 | 0.45 | -1.15 | 0.12 | -0.08 | 0.94 |

*Adjusted for age, gender, condition, disease duration, and pre-lockdown disease activity or emotional distress. Bold text indicates significant results.*

**Table 3. Adjusted regression coefficients for mental health.**

| | **PGA** | **p** | **Pain** | **p** | **Fatigue** | **p** |
|---|---|---|---|---|---|---|
| **Baseline in June** | | | | | | |
| Emotional distress | 0.21 | **<0.01*** | 0.24 | **<0.01*** | 0.36 | **<0.01*** |
| Depressive symptoms | 0.95 | **<0.01*** | 0.92 | **<0.01*** | 1.56 | **<0.01*** |
| Loneliness | 0.09 | 0.76 | 0.27 | 0.35 | 0.62 | 0.62 |
| **Follow-Up September** | | | | | | |
| Emotional distress | 0.15 | **0.01*** | 0.14 | **0.01*** | 0.14 | **0.02*** |
| Depressive symptoms | 0.69 | **0.01*** | 0.80 | **<0.01*** | 0.33 | **<0.01*** |
| Loneliness | 0.53 | 0.19 | 0.59 | 0.12 | 0.03 | 0.94 |
| **Follow-Up 5 November** | | | | | | |
| Emotional distress | 0.06 | 0.29 | -0.02 | 0.68 | 0.04 | 0.50 |
| Depressive symptoms | 0.65 | **0.04*** | 0.48 | 0.08 | 0.61 | **0.04*** |
| Loneliness | 0.53 | 0.24 | 0.32 | 0.41 | 0.61 | 0.14 |

*Adjusted for age, gender, condition, disease duration, and pre-lockdown disease activity or emotional distress. Bold text indicates significant results.*

*PGA: patient global assessment*

**Figure 1. Recruitment flow chart.**

**Figure 2. Changes from pre- to post-lockdown.**

PGA: patient global assessment.

**Figure 3. Changes to clinical care.**

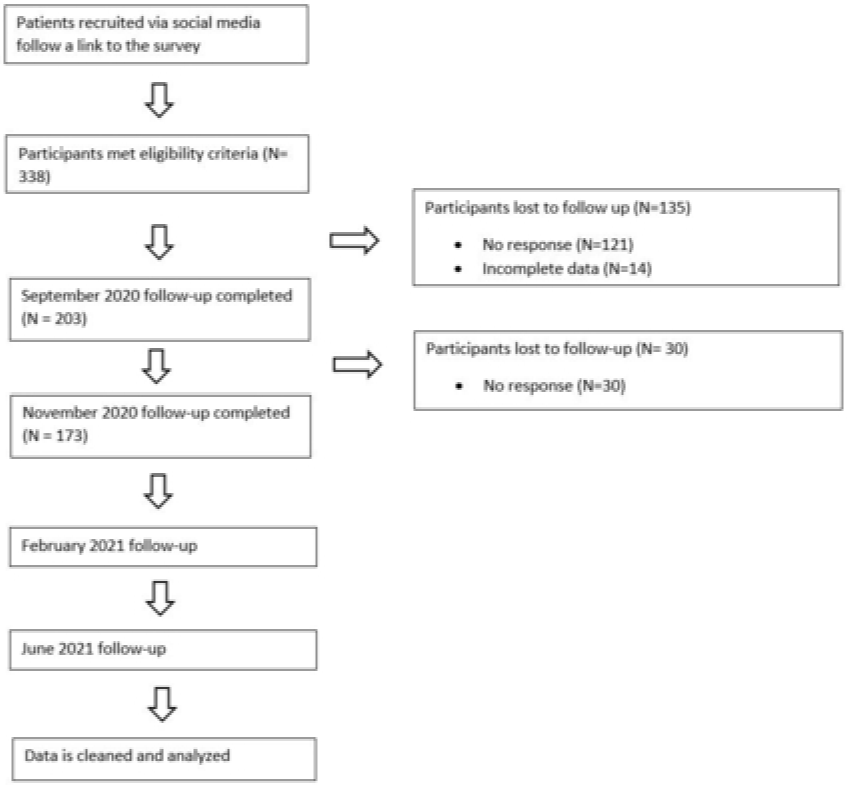

Figure 1. Recruitment Flow Chart

36x34mm (300 x 300 DPI)

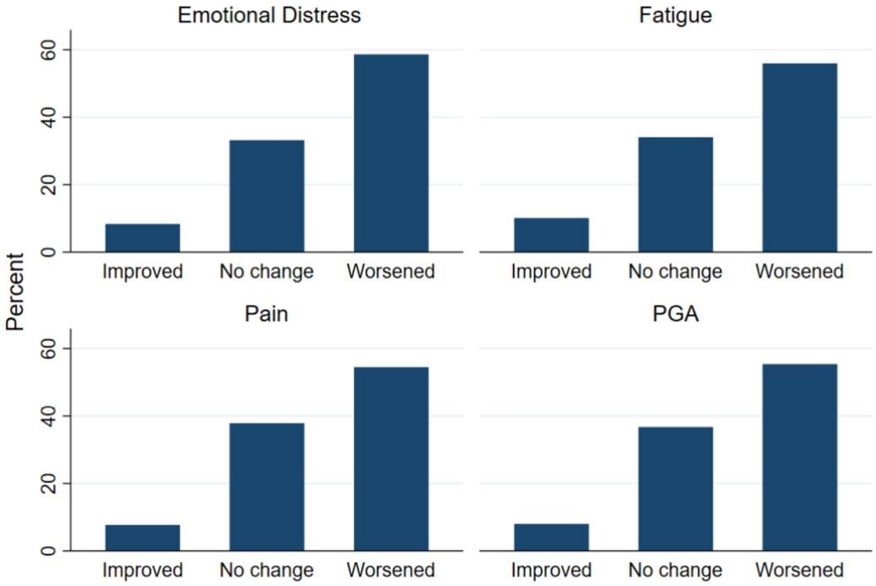

Figure 2. Changes from pre- to post-lockdown.
PGA: patient global assessment.

208x128mm (144 x 144 DPI)

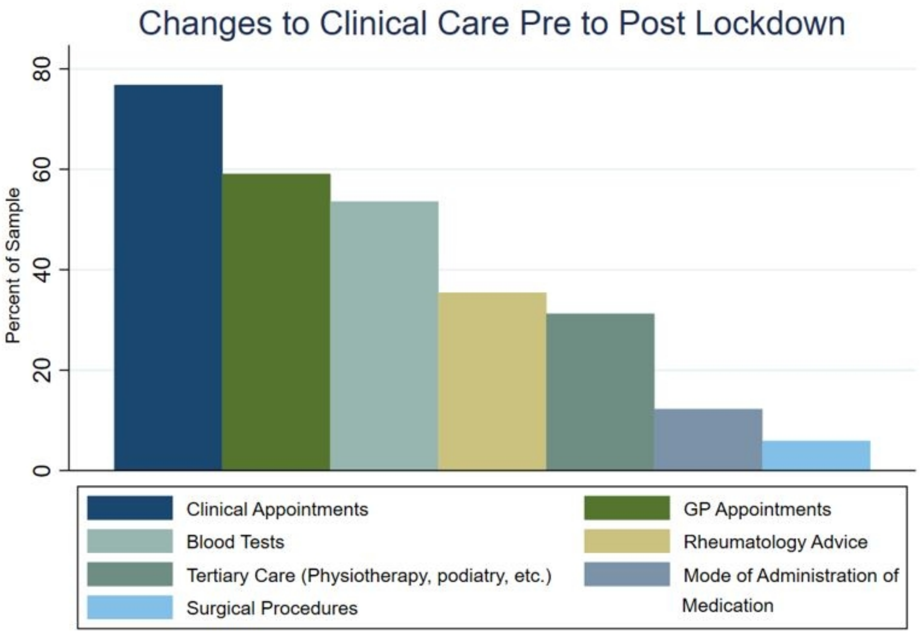

Figure 3. Changes to clinical care.

146x105mm (144 x 144 DPI)

## Supplementary Material

rkab095_Supplementary_Data

