# The impact of COVID-19 on clinical care, self-management and mental health of patients with inflammatory arthritis

**DOI:** 10.1093/rap/rkab095

**Published:** 2021-12-04

**Authors:** Melissa Sweeney, Lewis Carpenter, Savia de Souza, Hema Chaplin, Hsiu Tung, Emma Caton, James Galloway, Andrew Cope, Mark Yates, Elena Nikiphorou, Sam Norton

**Affiliations:** 1 Health Psychology Section, Institute of Psychiatry, Psychology and Neuroscience, King’s College London; 2 Centre for Rheumatic Diseases, King’s College London, Weston Education Centre, Cutcombe Road, London, UK

**Keywords:** inflammatory arthritis, lockdown, coronavirus disease 2019, clinical care, management, mental health, depression

## Abstract

**Objectives:**

The coronavirus disease 2019 (COVID-19) lockdown and ongoing restrictions in the UK affected access to clinical care, self-management and mental health for many patients with inflammatory arthritis. The aim of this study was to determine the impact of lockdown on inflammatory arthritis clinical care, self-management, disease outcomes and mental health.

**Methods:**

In total, 338 people with inflammatory arthritis participated in a prospective study, completing a series of online questionnaires. The questionnaires assessed demographics, inflammatory arthritis condition and management, clinical care, quality of life and mental health. Visual analogue scales (VASs) were completed at each assessment. Linear regression, controlling for confounders, was conducted to determine factors associated with physical and mental health outcomes.

**Results:**

More than half of participants reported worsening VAS by >10 points for patient global assessment (PGA), pain, fatigue and emotional distress during the initial lockdown. Changes in clinical care were associated with worse PGA (*b* = 8.95, *P* = 0.01), pain (*b* = 7.13, *P* = 0.05), fatigue (*b* = 17.01, *P* < 0.01) and emotional distress (*b* = 12.78, *P* < 0.01). Emotional distress and depression were also associated with worse outcomes in PGA, pain and fatigue, whereas loneliness was not. In contrast, physical activity seemed to mitigate these effects. Loneliness did not show any associations with outcomes. Over time, these effects decreased or disappeared.

**Conclusion:**

Changes to clinical care owing to lockdown were associated with worse disease outcomes in patients with inflammatory arthritis. There has also been a clear impact on mental health, with possibly complex relationships between mental health and psychosocial factors. Physical activity emerged as a key influence on disease outcomes and mental health.

Key messages

- The majority of patients reported worsened physical and mental health during lockdown.
- Changes in care and management were associated with worsening physical and mental health.
- The impact of lockdown changes on physical and mental health lessened over time.

## Introduction

Inflammatory arthritis is a collection of chronic autoimmune diseases that require ongoing pharmacological treatment and careful adherence to self-management behaviours [1, 2]. The coronavirus disease 2019 (COVID-19) lockdown in the UK from March to July 2020 disrupted clinical care and required a period of self-isolation for many patients [3]. Research into the impacts of changes to clinical care attributable to lockdown on disease outcomes of inflammatory arthritis patients were needed in the UK because disruptions to daily routines caused by lockdown and ongoing restrictions could potentially alter self-management behaviours and disease outcomes.

This would also be likely to impact mental health, given that worse disease activity has been shown to be associated with worse mental health in inflammatory arthritis [4, 5]. There is already some evidence that individuals with pre-existing physical or psychiatric co-morbidities appear to be at higher risk of mental health consequences from the pandemic [6]. Given that inflammatory arthritis patients already have higher rates of co-morbid mental health disorders compared with the general population [5, 7], they could be particularly vulnerable.

Finally, given that vulnerable inflammatory arthritis patients were advised to self-isolate for 12 weeks to reduce their risk of contracting COVID-19 (known as shielding), they could be at higher risk of mental health consequences from social isolation [3]. In the general population, quarantining was shown to be a risk factor for both short- and long-term negative psychological effects, such as increased rates of depression, insomnia, post-traumatic stress disorder and substance abuse [8]. Thus, research was needed into the effects of social isolation on the physical and mental health of inflammatory arthritis patients.

The objectives of this study were threefold: firstly, to evaluate how the COVID-19 lockdown from March to July 2020 impacted patients’ inflammatory arthritis symptoms, self-management and mental health in the short term during a period of easing of initial lockdown restrictions in June/July 2020; secondly, to evaluate the medium-term impacts on physical and mental health symptoms until November 2020; and thirdly, to determine the degree to which impacts on treatment and self-management were associated with worse physical and mental health symptoms in the short and medium term.

## Methods

### Design and recruitment

The IA-COVID study is a longitudinal mixed-methods study examining the impact of COVID-19 on the quality of life of people with inflammatory arthritis. Participants were recruited via social media and relevant charities. All participants provided written informed consent. Eligibility criteria were: aged ≥18 years, living in the UK and with an inflammatory arthritis condition. Although the eligibility criteria specified that respondents must be resident in the UK, three respondents from crown dependencies that form part of the British Isles but are not in the UK were included in the analyses. Ethical approval was obtained from King’s College London Research Ethics Committee (LRS-19/20-18186). Written informed consent was obtained from all participants. The study complies with the Declaration of Helsinki.

The data included in the present analysis consisted of the baseline data collected between 1 June and 3 July 2020, as lockdown restrictions in the UK were eased but shielding was ongoing. At the time of the baseline collection, shops re-opened, socializing with up to six people was allowed, and national travel resumed. Data from two additional follow-ups ∼3 months apart were also collected. The first follow-up collected data from early September 2020, during another period of looser restrictions that included working from home, a curfew, and a six-person limit on social gatherings. The second follow-up occurred in late November 2020, during a renewed period of strict restrictions, in which people were instructed to stay at home except for essential trips. Two more follow-ups were planned for February 2021 and June 2021; those data were not collected in time for the present study, but will be included in future analyses. Subsamples of participants also included an ecological momentary assessment study and a qualitative study [9].

### Measures

The questionnaires assessed various aspects of the impact of the COVID-19 pandemic and lockdown measures between 23 March and November 2020. Changes in these factors from before the lockdown were also evaluated.

The questionnaires were composed of the following full or shortened questionnaires: demographics, inflammatory arthritis condition, visual analogue scale (VAS) disease activity scale, VAS pain scale, VAS emotional distress scale, musculoskeletal health questionnaire (MSKHQ), personal health questionnaire depression scale (PHQ-8), generalized anxiety disorder assessment (GAD-7), University of California Los Angeles (UCLA) loneliness scale, Lubben social networks scale, healthy eating assessment, sleep questionnaire, global physical activity questionnaire (GPAQ) and the Capability Opportunity Motivation Behavior (COM-B) model. Some questions were modified to clarify them in the context of COVID-19. Additional researcher-designed questions were included regarding inflammatory arthritis management, changes to medication (beyond those recommended by the clinical care team), changes (yes/no) to various areas of clinical care, changes in self-management, co-morbidities, food shortages, social contact satisfaction, COVID-19 experience and symptoms, COVID-19 attitudes, fear of COVID-19, and the impact of COVID-19 on employment, finances and general wellbeing. Not all of these measures were used in the present analyses, but they might be used in sub-studies or future analyses.

### Disease outcome measures

The VASs were completed for the previous week, and all ranged from 0 to 100. The baseline study also retrospectively assessed pre-lockdown and early lockdown for the patient global assessment (PGA), pain and fatigue. VASs are considered appropriate to measure the intensity of an experience, such as distress or pain [10], and have been shown to have good validity and reliability [11, 12].

### Lifestyle measures

Diet was evaluated by a shortened healthy eating assessment measuring inflammatory diet patterns [13], which has good validity and sensitivity [14]. Higher scores indicated a more inflammatory diet. The baseline questions asked about the frequency of inflammatory and other foods eaten (fried/fast foods, sweets, sweetened beverages, fruit, vegetables, dairy, and red or processed meats) and also asked if they were eating less, the same or more of each item compared with before the COVID-19 measures.

Physical activity was measured at baseline with one question modified from the MSKHQ asking, ‘On how many days did you do a total of 30 min or more of physical activity, which was enough to raise your heart rate?’. Additionally, the baseline questionnaire asked if they engaged in less, the same or more physical activity compared with before the lockdown. The MSKHQ also shows good validity and reliability [15]. Finally, one researcher-designed question regarding changes to medication (yes/no to changes in dosage and/or frequency) was included.

### Quality of life and mental health measures

Emotional distress was measured with a VAS for the previous week in the baseline (post-lockdown) in June/July 2020 and both follow-up questionnaires. The baseline questionnaire also asked about emotional distress retrospectively for pre-lockdown (early March 2020) and peri-lockdown (April 2020). The PHQ-8 was used to measure depressive symptoms and has been validated in many contexts [16]. Two questions from the GAD-7 were used to assess anxiety: ‘Feeling nervous, anxious, or on edge’ and ‘Not being able to stop or control worrying’. The GAD-7 has shown good reliability and validity [17]. Several researcher-designed questions about psychosocial concerns were included. A shortened version of the UCLA loneliness scale using four questions relevant to lockdown context was used to assess loneliness in the baseline questionnaire. This scale has been established as a reliable and valid measure of loneliness [18]. One researcher-designed question assessed the level of fear or concern participants felt about COVID-19 (‘How concerned do you feel about COVID-19?’).

### Statistical analysis

Changes in mean VAS and S.d.s were calculated for PGA, pain, fatigue and emotional distress. Clinically meaningful improvement or worsening in each VAS score was considered as a change of ≥10 points from pre-lockdown to post-lockdown in June/July 2020 [19]. Repeated-measures ANOVAs were run for to determine whether there was any difference over time for PGA, pain, fatigue, emotional distress, diet, physical activity, depression, loneliness or fear of COVID-19. Additionally, Student’s paired *t*-tests were conducted to assess the difference between scores at the different time points. The percentage of the sample reporting better, same or worse outcomes compared with before lockdown on the VAS and the 95% CIs were calculated for PGA, pain, fatigue and emotional distress for pre- to post-lockdown scores, and for changes in clinical care and self-management behaviours. Violin plots of these changes in VAS scores were also produced. Demographics and key clinical characteristics were compared for participants who completed all surveys with those who dropped out.

Finally, linear regressions controlling for potential confounders of age, gender, condition, disease duration, pre-lockdown disease activity or emotional distress were conducted to determine the factors associated with worse outcomes on physical health measures and on mental health. Initially, baseline (June/July 2020) changes in clinical care, changes in medication, inflammatory diet and physical activity were used as predictors of PGA, pain, fatigue and emotional distress at baseline, September and November follow-ups. Changes in clinical care and medication were used as categorical predictors, where clinical care was coded as yes/no for each area of care that might have been affected, whereas medication changes were coded as yes/no but could include changes to either dosage or frequency. The remaining factors were used as continuous variables. Next, the baseline (June/July 2020) mental health factors of emotional distress, depression and loneliness were used as continuous predictors of PGA, pain and fatigue at baseline and September and November follow-ups. Models were completed separately for baseline, September and November outcomes. Effect sizes at all time points were calculated using omega-squared. All analyses were carried out using Stata (StataCorp LLC, v.16.0, Texas, USA).

## Results

**Fig. 1:**
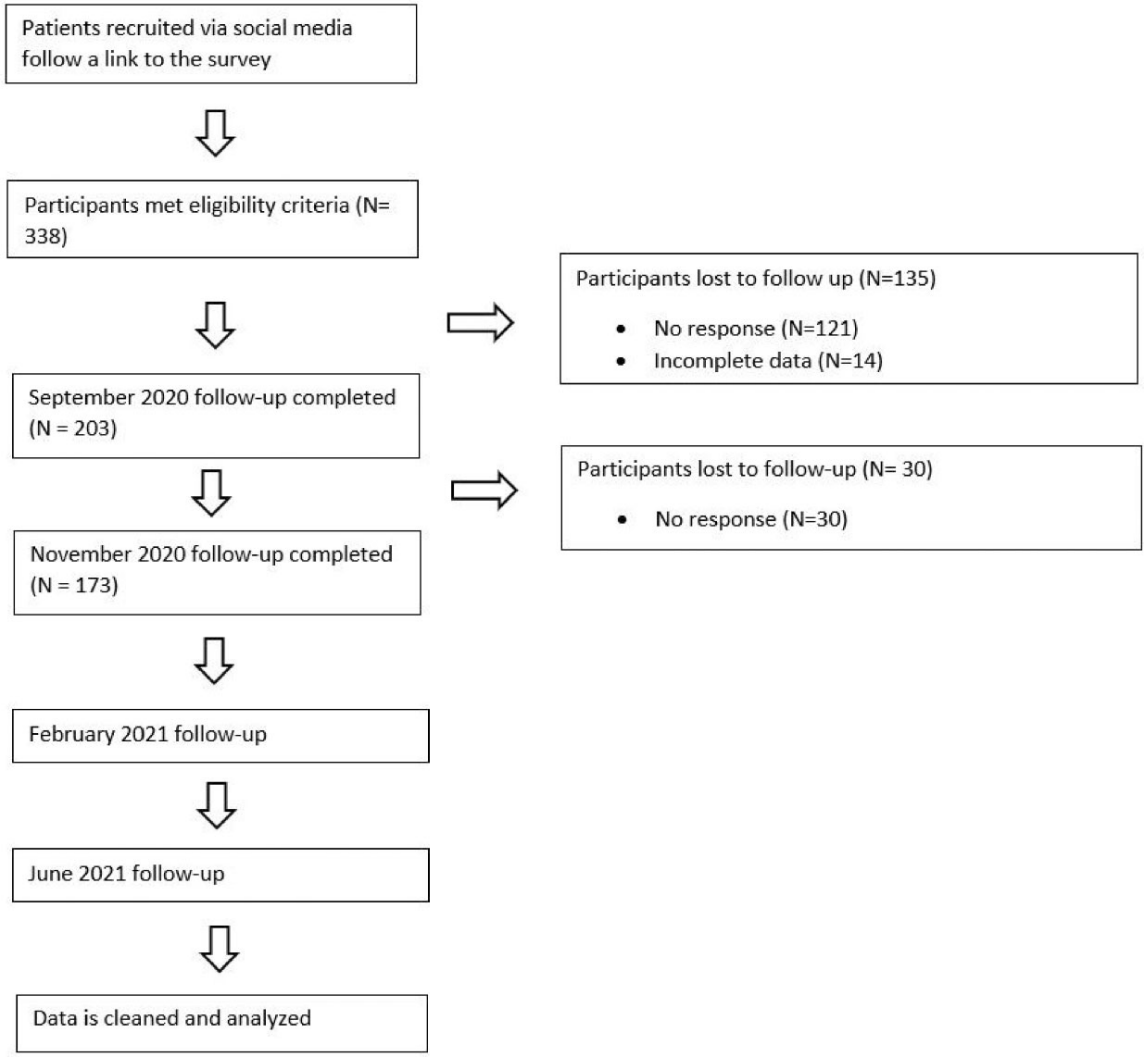
Recruitment flow chart

Table 1 summarizes the baseline characteristics of the sample by inflammatory arthritis condition. A total of 338 participants completed the baseline assessment in June. Data were available for 203 (60.0%) and 173 (51.2%) participants at the September and November follow-ups, respectively. The sample was largely female (90.2%) and White (97.5%), with an average age of 47.9 years (range 19–77 years). Fig. 1 shows a flowchart of the recruitment process. Those who completed all questionnaires were significantly older (*P* < 0.01) and had significantly higher scores on baseline pain (*P* < 0.01) compared with those who dropped out.

**Table 1:** Sample characteristics

Characteristic	Total sample	*P*-value	PsA	RA	Ankylosing spondylitis	Connective tissue disease	JIA
Total number of patients	338		98	100	50	85	5
Age, mean (s.d.), years	47.9 (13.6)		46.4 (12.0)	53.1 (14.4)	41.2 (12.1)	48.7 (12.7)	28.2 (11.5)
Gender, %							
Male	9.2		13.3	8	18	1.2	0
Female	90.2		85.7	92	82	97.7	100
Non-binary or other	0.6		1	0	0	1.2	0
Education, %							
No formal qualifications	3.6		3.1	2	4	5.9	0
O level, GCSE or equivalent	21.3		23.5	22	16	21.2	20
A level or equivalent	21		25.5	23	16	16.5	20
Undergraduate degree or equivalent	32.3		30.6	27	45	31.8	40
Postgraduate degree or equivalent	21.9		17.4	26	18	24.7	20
COVID infection, %							
Post-lockdown (June)	16.5		12.6	20.6	12.5	19.5	0
Follow-up 3 months (September)	12.43		12	18.5	5.6	8.2	0
Follow-up 5 months (November)	13.29		17.9	14.5	5.3	12.2	0
MSKHQ score, mean (s.d.)	33.9 (12.0)		35.5 (1.1)	31.3 (1.2)	37.5 (1.7)	32.7 (1.4)	39 (4.6)
Medication, %							
Biologics	44.7		52	55	72	89.4	80
Traditional conventional DMARDs	76.9		78.6	79	50	88.2	80
NSAIDs							
CSs	66.3		78.6	61	78	50.6	80
	50		46.9	53	48	51.8	40
Shielding, %	54.1		46.2	53.2	55.6	65	25
Patient global assessment, mean (s.d.)							
Pre-lockdown	44.5 (23.7)		47.8 (23.5)	41.4 (21.9)	53.0 (22.3)	38.7 (24.8)	56.4 (26.7)
Peri-lockdown	53.2 (24.7)	**<0.01**	55.5 (22.9)	50.6 (24.8)	61.9 (23.8)	48.5 (25.7)	52.4 (27.6)
Post-lockdown (June)	57.7 (25.3)	**<0.01**	60.2 (23.0)	53.8 (25.5)	67.9 (21.4)	52.9 (27.8)	66.2 (26.3)
Follow-up 3 months (September)	47.8 (25.6)	0.07	52.3 (25.6)	43.8 (24.7)	58.0 (23.7)	44.4 (26.0)	44.3 (38.8)
Follow-up 5 months (November)	48.5 (25.2)	0.04	53.5 (24.2)	43.9 (25.8)	51.7 (22.0)	47.1 (27.4)	52.5 (5.0)
Pain, mean (s.d.)							
Pre-lockdown	42.6 (25.6)		46.0 (26.5)	40.1 (23.7)	49.5 (26.0)	36.2 (24.7)	66.0 (27.0)
Peri-lockdown	51.1 (26.0)	**<0.01**	52.8 (25.0)	49.7 (26.5)	59.0 (25.0)	45.5 (26.1)	65.0 (27.0)
Post-lockdown (June)	56.7 (26.4)	**<0.01**	58.3 (25.0)	54.8 (27.0)	65.0 (23.5)	50.7 (27.9)	79.2 (9.3)
Follow-up 3 months (September)	46.8 (25.5)	0.03	53.1 (25.3)	40.1 (24.4)	54.5 (24.3)	44.3 (25.3)	73.7 (14.8)
Follow-up 5 months (November)	45.4 (24.8)	0.12	49.8 (24.8)	42.0 (26.2)	48.2 (22.0)	42.1 (24.4)	65.0 (12.9)
Fatigue, mean (s.d.)							
Pre-lockdown	46.9 (26.2)		59.5 (25.9)	42.3 (28.1)	49.4 (23.3)	46.6 (25.5)	45.6 (25.6)
Peri-lockdown	57.1 (25.8)	**<0.01**	61.4 (23.3)	51.2 (29.3)	59.8 (23.5)	58.1 (24.6)	48.2 (27.5)
Post-lockdown (June)	61.4 (26.5)	**<0.01**	65.1 (22.8)	55.2 (31.0)	65.4 (24.0)	61.7 (25.2)	63.8 (31.6)
Follow-up 3 months (September)	59.0 (26.2)	**<0.01**	61.8 (24.4)	53.1 (28.3)	68.9 (24.6)	59.2 (24.9)	70.0 (26.5)
Follow-up 5 months (November)	54.1 (25.6)	**<0.01**	57.4 (20.4)	47.8 (29.2)	58.5 (24.1)	56.5 (25.4)	51.0 (34.1)
Emotional distress, mean (s.d.)							
Pre-lockdown	31.0 (26.3)		32.2 (27.0)	27.7 (25.8)	35.1 (27.7)	31.0 (25.7)	29.2 (18.1)
Peri-lockdown	49.1 (29.1)	**<0.01**	48.9 (28.3)	45.7 (29.5)	58.0 (28.2)	47.5 (29.4)	56.0 (31.6)
Post-lockdown (June)	48.8 (29.2)	0.55	49.0 (28.7)	45.2 (28.8)	56.4 (27.8)	47.3 (29.7)	62.2 (42.0)
Follow-up 3 months (September)	38.9 (28.9)	**<0.01**	41.8 (27.7)	33.5 (28.9)	53.9 (26.7)	36.5 (29.2)	53.3 (25.2)
Follow-up 5 months (November)	39.8 (27.6)	**<0.01**	42.0 (26.6)	38.9 (31.3)	40.1 (24.1)	40.1 (26.1)	24.5 (17.9)
Inflammatory diet							
Pre-lockdown	—	—	—	—	—	—	—
Peri-lockdown	—	—	—	—	—	—	—
Post-lockdown (June)	8.8 (3.0)		9.3 (0.3)	8.5 (0.3)	8.9 (0.4)	8.4 (0.3)	8.5 (1.5)
Follow-up 3 months (September)	7.8 (2.4)	**<0.01**	8.3 (0.3)	7.2 (0.3)	9.4 (0.6)	7.7 (0.4)	7.3 (2.4)
Follow-up 5 months (November)	7.8 (2.5)	0.83	8.0 (0.4)	7.4 (0.4)	8.6 (0.5)	7.5 (0.4)	8.8 (1.4)
Physical activity							
Pre-lockdown	—	—	—	—	—	—	—
Peri-lockdown	—	—	—	—	—	—	—
							
Post-lockdown (June)	2.8 (2.1)		2.5 (0.2)	3.6 (0.3)	2.8 (0.3)	2.5 (0.2)	2.0 (1.0)
Follow-up 3 months (September)	3.1 (2.1)	0.79	2.3 (0.3)	3.7 (0.3)	3.4 (0.5)	3.2 (0.4)	2.0 (1.0)
Follow-up 5 months (November)	3.2 (2.2)	0.3	2.5 (0.3)	3.9 (0.3)	3.2 (0.6)	3.0 (0.4)	1.5 (0.3)
Depressive symptoms							
Pre-lockdown	—	—	—	—	—	—	—
Peri-lockdown	—	—	—	—	—	—	—
Post-lockdown (June)	10.5 (6.1)		11.0 (0.6)	8.9 (0.7)	12.0 (0.9)	10.9 (0.6)	12 (4.1)
Follow-up 3 months (September)	8.6 (5.9)	**<0.01**	9.8 (0.9)	7.1 (0.8)	12.5 (1.4)	7.9 (0.9)	9.3 (4.4)
Follow-up 5 months (November)	9.2 (5.9)	**<0.01**	10.6 (0.9)	7.4 (0.8)	10.6 (1.3)	9.8 (0.9)	8 (2.9)
Loneliness							
Pre-lockdown	—	—	—	—	—	—	—
Peri-lockdown	—	—	—	—	—	—	—
Post-lockdown (June)	10.3 (4.0)		10.2 (0.4)	10.4 (0.4)	10.8 (0.6)	10.1 (0.5)	11.0 (2.1)
Follow-up 3 months (September)	9.9 (3.8)	**<0.01**	10.0 (0.6)	9.7 (0.5)	12.1 (0.8)	8.9 (0.6)	13 (1.5)
Follow-up 5 months (November)	9.3 (4.0)	**0.04**	9.1 (0.6)	9.3 (0.6)	9.9 (0.9)	9.3 (0.7)	8.8 (1.3)
COVID fear							
Pre-lockdown	—	—	—	—	—	—	—
Peri-lockdown	—	—	—	—	—	—	—
Post-lockdown (June)	3.8 (1.0)		3.7 (0.1)	3.7 (0.1)	4.0 (0.1)	3.7 (0.1)	4.0 (1.0)
Follow-up 3 months (September)	3.4 (1.0)	**<0.01**	3.4 (0.2)	3.4 (0.2)	4.0 (0.2)	3.3 (0.2)	3.7 (0.3)
Follow-up 5 months (November)	3.5 (1.0)	**0.01**	3.5 (0.1)	3.5 (0.1)	4 (0.2)	3.6 (0.2)	2.5 (0.3)

Bold text indicates significant results. MSKHQ: musculoskeletal health questionnaire.

Repeated-measures ANOVA found differences over time for PGA [*F*(4, 1036) = 34.58 *P* < 0.01], pain [*F*(4, 1036) = 40.54, *P* < 0.01], fatigue [*F*(4, 1035) = 43.11, *P* < 0.01], emotional distress [*F*(4, 1012) = 55.67, *P* < 0.01], diet [*F*(2, 273) = 10.88, *P* < 0.01), depression [*F*(2, 286) = 8.43, *P* < 0.01], loneliness [*F*(2, 281) = 5.97, *P* < 0.01] and fear of COVID-19 [*F*(2, 270) = 8.26, *P* < 0.05]. Physical activity was not significantly different across time points [*F*(2, 262) = 0.15, *P* = 0.86]. Student’s paired *t*-tests are shown in Table 1 identifying the time points with significant differences.

### Physical health

**Fig. 2:**
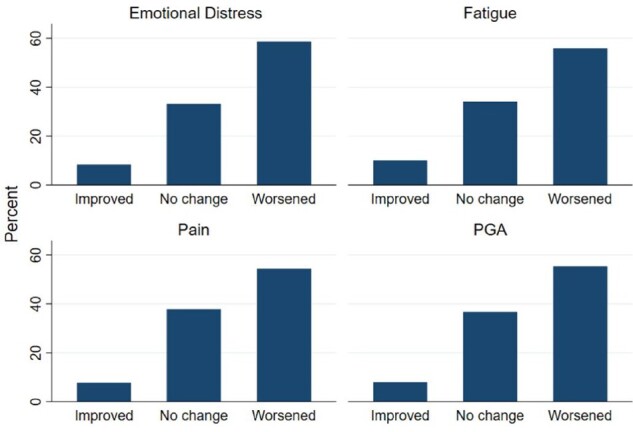
Changes from pre- to post-lockdown PGA: patient global assessment.

The mean VAS scores and S.d.s during pre-, peri- and post-lockdown from baseline and the September and November follow-ups are displayed in Table 1. On average, all measures of disease activity (PGA, fatigue and pain) showed worsening from pre-lockdown (February 2020) to post-lockdown (June 2020) (Fig. 2; Supplementary Fig. S1, available at *Rheumatology Advances in Practice* online.). In contrast, emotional distress was highest in peri-lockdown. The majority of the overall sample reported worsening outcomes during the lockdown for all disease measures; however, the results were mixed, and many participants also reported that their disease activity stayed the same, while a minority reported improvements.

At the follow-ups, the VAS scores for PGA, pain, fatigue and emotional distress had improved relative to the end of the lockdown in June, but remained higher than pre-lockdown levels. Pain and fatigue VAS scores showed consistent trends downward over time after the lockdown, whereas PGA and emotional distress showed a slight increase again in November.

Demographic and earlier clinical measurements were examined for associations with later physical outcomes. None of the demographic characteristics was predictive of physical health outcomes at baseline or the follow-ups except for duration of the inflammatory arthritis condition, which was significantly associated with PGA in September (*b* = 0.003, *P* < 0.01). The pre- and post-lockdown measurements of PGA, pain and fatigue were significantly associated with all their respective measurements at baseline and/or follow-ups, with the exception of PGA pre-lockdown, which was not significantly associated with PGA in November.

### Clinical care

**Fig. 3:**
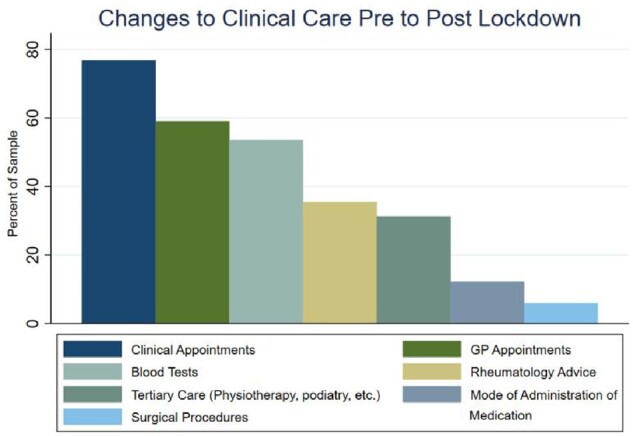
Changes to clinical care

Overall, 87.45% of participants experienced change to their clinical care (as indicated in Fig. 3), with the greatest impact on clinical appointments (76.8%), general practioner appointments (59.1%) and blood tests (53.6%). A detailed breakdown of the percentage [95% CI] of participants with any changes in each of the clinical care areas during the lockdown is provided in Supplementary Table S1, available at *Rheumatology Advances in Practice* online.

Linear regression analyses (Table 2) demonstrate that those reporting changes to clinical care at baseline had significantly worse PGA (*b* = 8.95, *P* = 0.01), pain (*b* = 7.13, *P* = 0.05), fatigue (*b* = 17.01, *P* < 0.01) and emotional distress (*b* = 12.78, *P* < 0.01) at baseline, even when controlling for pre-lockdown levels of the outcome. Results remained significant when controlling for fear of COVID-19 and COVID-19 infection status. The Omega squared (*w*^2^) effect size of changes to clinical care was small for PGA (*w*^2^ = 0.02) pain (*w*^2^ = 0.01) and emotional distress (*w*^2^ = 0.03), whereas it was medium for fatigue (*w*^2^ = 0.07).

**Table 2:** Adjusted regression coefficients for clinical care and lifestyle

Clinical care and lifestyle	PGA	*P*-value	Pain	*P*-value	Fatigue	*P*-value	Emotional distress	*P*-value
Baseline in June								
Changes to clinical care	8.95	**0.01**	7.13	**0.047**	17.01	**<0.01**	12.78	**<0.01**
Changes to medication	13.13	**<0.01**	11.47	**<0.01**	14.83	**<0.01**	7.43	0.12
Inflammatory diet	0.61	0.10	0.72	0.19	0.99	**0.02**	0.88	0.08
Physical activity	−2.40	**<0.01**	−2.43	**<0.01**	−2.50	**<0.01**	−2.41	**<0.01**
Follow-up September								
Changes to clinical care	5.34	0.29	7.96	0.09	10.76	**0.04**	5.88	0.30
Changes to medication	2.45	0.65	1.97	0.70	3.09	0.57	0.24	0.97
Inflammatory diet	−0.33	0.53	−0.27	0.58	0.18	0.73	−0.36	0.55
Physical activity	−1.25	0.11	−1.94	**0.01**	−19.10	**0.02**	−0.61	0.48
Follow-up November								
Changes to clinical care	1.02	0.84	5.70	0.19	−2.02	0.66	−2.66	0.67
Changes to medication	7.35	0.20	−1.50	0.76	6.39	0.21	7.64	0.29
Inflammatory diet	1.78	**<0.01**	0.19	0.73	0.34	0.54	0.90	0.24
Physical activity	−1.59	0.05	−0.53	0.45	−1.15	0.12	−0.08	0.94

Results are adjusted for age, gender, condition, disease duration, and pre-lockdown disease activity or emotional distress. Bold text indicates significant results.

At the follow-ups, the impact of clinical care changes remained significant only for fatigue in September (*b* = 10.76, *P* = 0.04), but was no longer significant by November. The effect size for fatigue decreased over time, with it having faded to a small effect size in September (*w*^2^ = 0.02) compared with the medium effect size at baseline. None of the other outcomes of PGA, pain and emotional distress remained significant over time at the follow-ups.

Table 2 shows that, overall, the majority of participants (89.7%) reported not altering their medication during the lockdown period at baseline. Table 2 also shows the linear regressions for changes in medication, adjusted for pre-lockdown levels of outcomes. Medication non-adherence at baseline was also significantly associated with worse PGA (*b* = 13.12, *P* < 0.01), pain (*b* = 11.47, *P* < 0.01) and fatigue (*b* = 14.83, *P* < 0.01) but not emotional distress (*b* = 7.43, *P* < 0.12) at baseline. Effect sizes for changes to medication were small for PGA (*w*^2^ = 0.04), pain (*w*^2^ = 0.03) and fatigue (*w*^2^ = 0.04). None of these effects was still significant at the follow-ups in September or November.

When the analyses were repeated with only participants who completed all questionnaires, changes to clinical care were no longer significant at baseline for emotional distress, nor were they significant in September for pain or fatigue, but they were significant in November for pain (*b* = 16.9, *P* = 0.05). Changes in medication were no longer significant at baseline for fatigue.

### Lifestyle

More than half (64.3%) of the participants reported making changes to their diet during the lockdown, and 51.1% reduced their physical activity. Table 2 displays the regression coefficients for self-management behaviours as predictors of disease outcomes. An inflammatory diet was significantly associated with fatigue only (*b* = 0.99, *P* = 0.02), whereas physical activity was associated with PGA (*b* = −2.40, *P* < 0.01), pain (*b* = −2.43, *P* < 0.01), fatigue (*b* = −2.5, *P* < 0.01) and emotional distress (*b* = −2.41, *P* < 0.01) in June. The results remained significant when controlling for fear of COVID-19 and COVID-19 infection status. The effect sizes of physical activity were medium for PGA (*w*^2^ = 0.07), pain (*w*^2^ = 0.07), fatigue (*w*^2^ = 0.06) and emotional distress (*w*^2^ = 0.04).

At the follow-ups, physical activity at baseline remained significantly associated with pain (*b* = −1.94, *P* = 0.01) and fatigue (*b* = −19.1, *P* = 0.02) in September, and by November none of the effects remained. The effect sizes for physical activity also decreased over time for pain (*w*^2^ = 0.04) and fatigue (*w*^2^ = 0.03) by September compared with baseline. An inflammatory diet was no longer significant for fatigue at follow-ups. However, although inflammatory diet was not significantly associated with PGA at baseline in June, it was significantly associated with PGA in November (*b* = 1.78, *P* < 0.01, *w*^2^ = 0.05), indicating a delayed effect. When the regressions were repeated with the sample including only those who completed all questionnaires, diet was significant at baseline for PGA (*b* = 2.25, *P* = 0.01) and pain (*b* = 2.26, *P* = 0.02). However, physical activity was no longer significant for pain at the September follow-up.

### Mental health

The majority (58.6%) of participants in the overall sample reported that their emotional distress worsened during the lockdown, although the changes were mixed (Supplementary Fig. S1, available at *Rheumatology Advances in Practice* online). This pattern was similar across conditions, with the exception of JIA, but this group had a sample size of only five.

Table 3 shows that emotional distress at the end of the lockdown was found to be significantly associated with PGA (*b* = 0.21, *P* < 0.01), pain (*b* = 0.24, *P* < 0.01) and fatigue (*b* = 0.36, *P* < 0.01). Likewise, depression was associated with all the disease activity outcomes in June, at the end of lockdown: PGA (*b* = 0.95, *P* < 0.01), pain (*b* = 0.92, *P* < 0.01) and fatigue (*b* = 1.56, *P* < 0.01). Loneliness was not associated with any of the disease activity outcomes. The results remained significant when controlling for fear of COVID-19 and COVID-19 infection status.

**Table 3:** Adjusted regression coefficients for mental health

Mental health	PGA	*P*-value	Pain	*P*-value	Fatigue	*P*-value
Baseline in June						
Emotional distress	0.21	**<0.01**	0.24	**<0.01**	0.36	**<0.01**
Depressive symptoms	0.95	**<0.01**	0.92	**<0.01**	1.56	**<0.01**
Loneliness	0.09	0.76	0.27	0.35	0.62	0.62
Follow-up in September						
Emotional distress	0.15	**0.01**	0.14	**0.01**	0.14	**0.02**
Depressive symptoms	0.69	**0.01**	0.80	**<0.01**	0.33	**<0.01**
Loneliness	0.53	0.19	0.59	0.12	0.03	0.94
Follow-up in November						
Emotional distress	0.06	0.29	−0.02	0.68	0.04	0.50
Depressive symptoms	0.65	**0.04**	0.48	0.08	0.61	**0.04**
Loneliness	0.53	0.24	0.32	0.41	0.61	0.14

Results are adjusted for age, gender, condition, disease duration, and pre-lockdown disease activity or emotional distress. Bold text indicates significant results. PGA: patient global assessment.

At the follow-ups, emotional distress remained significant for PGA (*b* = 0.15, *P* = 0.01), pain (*b* = 0.14, *P* = 0.01) and fatigue (*b* = 0.14, *P* = 0.02) in September. Depression also remained significant in September for PGA (0.65, *P* = 0.04), pain (*b* = 0.48, *P* = 0.08) and fatigue (*b* = 0.61, *P* = 0.04), and in November it was significant only for PGA (*b* = 0.65, *P* = 0.04) and fatigue (*b* = 0.61, *P* = 0.04).

When the regressions were repeated with the sample including only those who completed all questionnaires, emotional distress was no longer significant in the September follow-up for PGA, pain or fatigue, and depression was no longer significant for PGA and fatigue. In November, depression was no longer significant for PGA or fatigue. None of the demographic characteristics was significantly associated with emotional distress at baseline or the follow-ups. The pre-lockdown and post-lockdown measurements of emotional distress were significantly associated with the later measurements of emotional distress at baseline and follow-ups.

The effect sizes for emotional distress were large for PGA (*w*^2^ = 0.10), pain (*w*^2^ = 0.12) and fatigue (*w*^2^ = 0.23). For depression, the effect sizes were medium for PGA (*w*^2^ = 0.09) and pain (*w*^2^ = 0.07) and large for fatigue (*w*^2^ = 0.17). The effect size for social contact on pain was small (*w*^2^ = 0.02). The effect sizes for emotional distress were reduced at the follow-up in September (PGA, *w*^2^ = 0.03; pain, *w*^2^ = 0.03; and fatigue, *w*^2^ = 0.02). For depression, the effect sizes were also reduced at follow-up in both September (PGA, *w*^2^ = 0.03; pain, *w*^2^ = 0.05; and fatigue, *w*^2^ = 0.04) and November (PGA, *w*^2^ = 0.02; and fatigue, *w*^2^ = 0.02).

## Discussion

Patients with inflammatory arthritis experienced significant disruptions to their clinical care, lifestyle and mental health during the COVID-19 lockdown and ongoing restrictions in 2020. These changes were associated with worse disease activity, indicating that clinicians should be aware of the adverse effects of changes to clinical care and consider ways to mitigate the negative effects.

Changes to lifestyle behaviours during the lockdown varied widely among patients. The mixed results for inflammatory diet over time could indicate differing short- and long-term mechanisms, such as different inflammatory pathways or causes. Changes in physical activity were also mixed, reflecting results in other studies [20, 21]. However, given that physical activity had a larger impact on disease activity measures than changes in medication and clinical care in the long term, its importance in inflammatory arthritis self-management and future interventions is underscored. Physical activity might also offset some of the impacts of disruptions to clinical care; therefore, clinicians should continue to support patient education around it [23, 24]. The qualitative sub-study associated with the present study provides further insight into explanations for changes in behaviour [9].

It has already been established that, outside of lockdowns, emotional distress is intertwined with worse disease outcomes [25–27]. Our results suggest that this is consistent under lockdown too [28]. Other research has indicated that mental health concerns have increased during the pandemic, suggesting that mental health might be of increased importance during this time [29–31]. This should prompt professionals to prioritize access to mental health resources to prevent emotional distress from affecting inflammatory arthritis outcomes.

The null results for loneliness might be indicative of the overlap between different aspects of mental health and psychosocial factors. Although the present study did not find loneliness to be associated directly with physical health outcomes, other research has indicated that loneliness has worsened during lockdowns and has been associated with depression and suicidality [32, 33]. Additionally, the UCLA loneliness scale is a common measure, but has not been validated in the context of lockdowns and should therefore be interpreted with caution.

This study had the benefit of a large sample size, although it appears to have some bias in gender, age and ethnicity. The study was also limited in that the pre-lockdown and peri-lockdown measures from baseline were retrospective self-report up to several months prior. The analyses included descriptive statistics of all the retrospective measures, but the regressions were limited to more recent measures (last 2 weeks), which would be more reliable. Also, some of the questions were shortened from existing scales, modified to fit the context of COVID-19, or researcher designed in the absence of pre-existing scales relevant to COVID-19. These questions would also be a limitation because they were not validated.

The present analyses suggest the impacts of lockdown show a general decrease over time. Given that there are further follow-up questionnaires from February 2021 and June 2021, future analyses can potentially examine whether these decreases continue over longer periods of time.

Lastly, this study suggests that professionals should consider the adverse effects on patients of changes to care and lifestyle owing to the COVID-19 lockdown and restrictions, because these changes are associated with worsening of disease outcomes and mental health. Additionally, the decrease in the impacts over time indicates that more support during initial phases of lockdowns, followed by gradual easing, could be most appropriate. Guided by insights from this study, professionals have the potential to improve patient support in the future and prevent adverse impacts on patient outcomes.

## Supplementary Material

rkab095_Supplementary_Data

## Data Availability

The data underlying this article will be shared on reasonable request to the corresponding author.
