# Supplementary material for: The impact of COVID-19 on clinical care, self-management and mental health of patients with inflammatory arthritis
Source: Rheumatol Adv Pract. 2021 Dec 4;6(1):rkab095. doi: 10.1093/rap/rkab095 (PMC8690299; doi:10.1093/rap/rkab095)
Supplement: rkab095_Supplementary_Data [file rkab095_supplementary_data.docx]

**Supplementary Material**

**Supplementary Table S1. Changes in disease outcomes and lifestyle**

|  | **PERCENT CHANGES [95% CI], N** |
| --- | --- |
| **PGA**  Better  Same  Worse  **Pain**  Better  Same  Worse  **Fatigue**  Better  Same  Worse  **Emotional Distress**  Better  Same  Worse | 8.0 [5.3-11.4]  36.7 [31.5-42.1]  55.3 [49.9-60.7]  7.7 [5.1-11.1]  37.9 [32.7-43.3]  54.4 [49.0-59.8]  10.1 [7.1-13.8]  34.0 [29.0-39.4]  55.9 [50.4-61.3]  8.3 [5.6-11.8]  33.1 [28.1-38.4]  58.6 [53.1-63.9] |
| **Care Access**  Any changes  No changes  Clinical appointments  GP appointments  Blood tests  Rheumatology advice  Tertiary care  Surgical  Mental health care | 87.5 [82.9-91.2], N=237  10.1 [7.1-13.8], N=34  76.8 [70.9-82.0], N=182  59.1 [52.5-65.4], N=140  53.6 [47.0-60.1], N=127  35.4 [29.4-41.9], N=84  31.2 [25.4-37.5], N=74  5.9 [3.3-9.7], N=14  8.9 [6.1-12.4], N=30 |
| **Medication**  No changes  Any changes  Reduced dose  Reduced frequency  Reduced both dose & frequency  Stopped any medication  Access to medications  Mode of administration of medication | 89.7 [85.4-93.2], N=243  10.3 [7.0-14.6], N=28  32.1 [15.9-52.4], N=9  35.7 [18.6-55.9], N=10  3.6 [0.1-18.4], N=1  28.6 [13.2-48.7], N=8  17.2 [13.3-21.6], N=58  12.2 [8.4-17.1], N=29 |
| **Diet due to food shortages**  None  Slight  Significant | 37.9 [32.2-43.8]  54.6 [48.6-60.6]  7.5 [4.7-11.2] |
| **Inflammatory Diet**  Any changes  No changes | 64.3 [58.4-69.9]  35.7 [30.1-41.6] |
| **Physical Activity**  More  Same  Less | 19.1 [14.6-24.2], N=53  29.9 [24.5-35.6], N=83  51.1 [45.0-57.1], N=142 |

*^a^PGA: Patient Global Assessment; ^b^GP: General Physician*

**Supplementary Figure S1. Violin plots indicating the distribution of changes in VAS scores from pre- to post-lockdown**


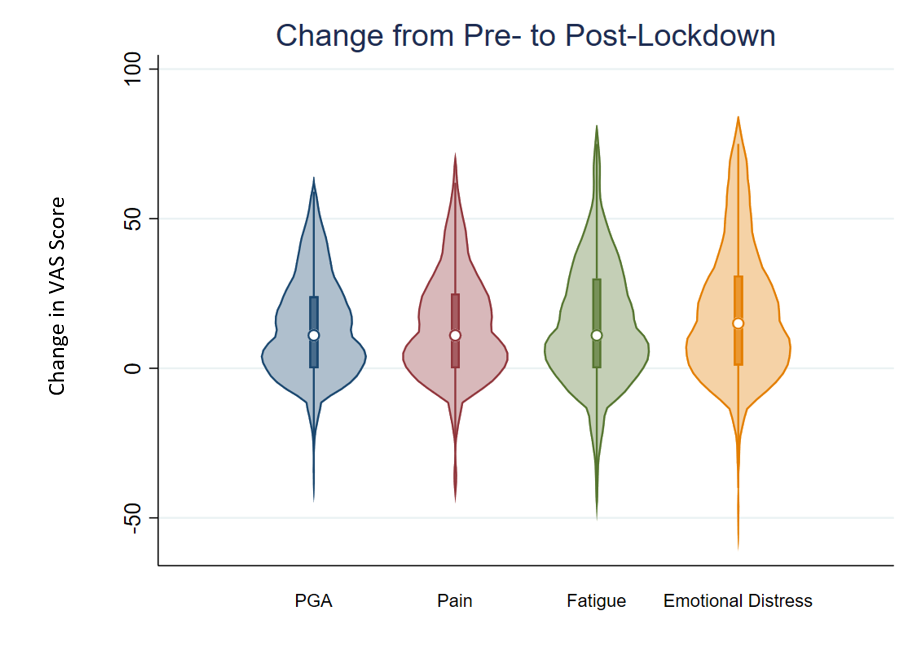


*^a^PGA: Patient Global Assessment; ^b^VAS: Visual Analogue Scale*
